# A Mathematical Model for the Electrical Resistivity of Cement Paste at Early Ages Considering the Partially Saturated State

**DOI:** 10.3390/ma13153306

**Published:** 2020-07-24

**Authors:** Ye Tian, Xin Xu, Haodong Ji, Zushi Tian, Xianyu Jin, Nanguo Jin, Dongming Yan, Shengwen Tang

**Affiliations:** 1 Department of Civil Engineering and Architecture, Zhejiang University, Hangzhou 310058, China; cetianye@zju.edu.cn (Y.T); 21912155@zju.edu.cn (X.X.); jihd@zju.edu.cn (H.J.); xianyu@zju.eu.cn (X.J.); jinng@zju.edu.cn (N.J.); dmyan@zju.edu.cn (D.Y.); 2 Department of Water Resources and Hydropower Engineering, Wuhan University, Wuhan 430070, China; tangsw@whu.edu.cn

**Keywords:** cement paste, hydration degree, electrical resistivity, temperature, partially saturated

## Abstract

For cementitious materials, electrical resistivity is often used in the study of the cement hydration process at early age, as one of the few indicators that can be continuously and non-destructively monitored. Variation characteristics of resistivity are widely reported to interact with the early-age performance of cement paste, such as hydration kinetics parameters and setting time. However, there is no reasonable mathematical model to predict the resistivity at early ages, especially within the first 24 h, due to significant changes in the porosity and degree of saturation. In this work, a mathematical model was developed by considering the partially saturated state and density change of C-S-H (calcium silicate hydrate). To verify the model, two experimental methods were chosen, including the non-contact electrical resistivity test and isothermal calorimetry test. The hydration heat and resistivity of cement paste with a water–cement ratio of 0.35 and 0.45 were continuously monitored for 3 days. In the resistivity test, embedded temperature sensors were used to monitor the internal temperature and temperature correction was treated carefully in order to obtain accurate data. The test results prove that the mathematical model can accurately predict electrical resistivity and describe the saturation state of early-age cement pastes under sealed curing.

## 1. Introduction

The long-term performance of cementitious materials is determined by the early cement hydration process. It is well known that the cementitious material system is in an unsteady state and its macroscopic characteristics and microstructure change quickly at the early stage of hydration, especially within the first 48 h. Experimentally, there are only a few methods, which can accurately and continuously monitor the properties of cementitious materials by non-destructive means, such as isothermal calorimetry and electrical resistivity monitors. Isothermal calorimetry measures the hydration heat to characterize the hydration kinetics [1]. A detailed investigation suggested that the hydration process of cement paste can be divided into three phases, as the nucleation and growth reactions phase, boundary reactions phase and governs reactions phase [2,3]. In comparison to isothermal calorimetry, electrical resistivity monitors [4,5] are widely applied in the study of cement hydration because they reveal more information on the hydration mechanism [6,7,8,9,10,11] and microstructure evolution [12,13,14,15]. Based on the evolution of resistivity, the cement hydration process can be divided into four periods: dissolution, induction, accelerated and decelerated periods [6,7]. A series of investigations were performed to further discuss the early-age properties of cementitious materials, such as the initial setting time [10,16,17], strength [11,17,18,19], hydration kinetic parameters [20,21] and chemical shrinkage [22,23], through electrical resistivity [24,25,26,27,28].

However, the existing cement hydration models do not explain the experimental data of isothermal calorimetry and electrical resistivity monitors at very early ages (less than 24 h), though they work well on a time scale of days and weeks. Firstly, it is difficult to characterize pore microstructure, pore solution distribution, and the degree of saturation at early age. Some common experimental techniques request dry samples, such as mercury intrusion porosimetry (MIP) and nitrogen adsorption isotherms (NAI). The process of hydration termination and drying may cause the damaging of pore structure, which is fragile at early age. Furthermore, the MIP technique always underestimates small pores in the cementitious material. In recent years, nuclear magnetic resonance (NMR), as a non-destructive monitoring technology, has been utilized to detect and distinguish capillary water gel water and air in cementitious materials [29,30]. On the basis of NMR data, an innovative analytical cement hydration model was proposed [31], which reveals the change of C-S-H gel density during hydration. The model is more reasonable and accurate in comparison with the widely-used Power’s model [32] that assumes C-S-H gel density as a constant. Secondly, how the pore solution existing in different ways contributes to the overall conductivity needs to be further determined. Capillaries are generally considered to have good connectivity but the gel pore solution, which exists in the form of small isolated capillaries, micropores and interlayer space in both low density (LD) C-S-H gel and high density (HD) C-S-H gel [33,34], is separated by the gel chain. Finally, the cement paste is in a typical partially saturated state under sealed curing conditions, where water is consumed continuously by cement hydration. The degree of saturation has a significant effect on the overall resistivity [35,36].

In this work, a mathematical model was developed to predict the electrical resistivity of cement paste at early ages. In the model, physical and chemical changes of cement hydration at early ages were considered, including the partially saturated state and density change of C-S-H. The hydration heat and resistivity of cement paste with a water–cement ratio of 0.35 and 0.45 were continuously monitored for 3 days utilizing the non-contact electrical resistivity test and isothermal calorimetry test. After careful temperature correction, the test data were used to verify the rationality and accuracy of the model. It helps to better understand the relationship of the pore structure, moisture content, and resistivity of cement paste at early ages, especially within the first 48 h.

## 2. A Mathematical Model of Electrical Resistivity

### 2.1. Correction of Temperature Effect

Temperature affects two aspects of the resistivity of cement paste, electrical measurement and hydration [5,37]. The former is a single physical mechanism, that is, temperature influences the measured values of resistivity since the pore solution resistivity becomes lower at higher temperatures. The latter is a chemical mechanism, that is, the hydration rate of cementitious materials increases as the reaction temperature rises.

For the effect of temperature on electrical measurement, previous investigations [38,39,40] used a linear equation with the temperature correction coefficient for cementitious materials covering a range of 0.018 to 0.025 °C^−1^. The value is close to the temperature correction coefficient of pure water and alkaline solutions [41]. However more recently, investigations [4,42,43,44] suggested the use of the activation-energy-based formula to correct the temperature effect presented in Equation (1):

(1) $$\rho_{T_{0}}=\rho_{T}\cdot \exp\left[\frac{E_{a-cond}}{R}\left(\frac{1}{T}-\frac{1}{T_{0}}\right)\right],$$

where *E_a-cond_* is the activation energy of conduction, R is the gas constant and the value is 8.31 J/(K·mol). Previous investigations reported different values for the activation energy of conduction of cementitious materials including 18.7, 19.9–23.4, 16.8–21.2, 16–30 kJ/mol [4,5,43,44]. Moreover, the activation energy of conduction is related to relative humidity and curing conditions [5,45]. Therefore, the value of 23.4 kJ/mol measured in sealed conditions [5] was chosen in this research.

For the effect of temperature on hydration, the equivalent maturity theory is usually used to correct the resistivity to the value at the reference temperature. According to the theory, time *t* at the variable temperature *T* of actual reaction corresponds to the equivalent maturity age *t_eq_* at reference temperature *T_ref_*. That means the hydration degree, as well as the resistivity, at *t* is equal to that at *t_eq_* under a constant reference temperature *T_ref_*. Therefore, the following equation can be given as:

(2) $$\rho_{T_{ref}}\left(t_{eq}\right)=\rho_{T_{var}}\left(t\right),$$

where $\rho_{T_{ref}}$ is the resistivity of cement paste under reference temperature *T_ref_* at the equivalent maturity age *t_eq_*, $\rho_{T_{var}}$ is the resistivity at the variable temperature *T* of actual reaction at the actual maturity age *t*. The equivalent maturity age *t_eq_* can be calculated by the Arrhenius equation [46,47], as:

(3) $$t_{eq}=\int_{0}^{t}\exp\left[\frac{E_{a-hyd}}{R}\left(\frac{1}{T_{ref}}-\frac{1}{T\left(t\right)}\right)\right]dt,$$

where *E_a-hyd_* is the activation energy of hydration reaction. It should be noted that the equivalent time is affected by the temperature history changing with time rather than the temperature at a certain moment or the average temperature. For ordinary Portland cement, the activation energy is generally obtained within a narrow range from 40 to 45 kJ/mol, as recommended by ASTM C 1074. In this study, the value of 40.5 kJ/mol was adopted [48].

### 2.2. A Mathematical Model of Resistivity

The relationship of the overall resistivity of composite materials and the resistivity of each phase can be described by the general effective media (GEM) model [49,50], and be calculated by the following equations for cementitious systems [12], as:

(4) $$\varphi_{low}=\left[\left(1-\varphi_{c}\right)F^{-\frac{1}{m}}-\varphi_{c}\right]\cdot \left(\frac{M^{\frac{1}{m}}-F^{-\frac{1}{m}}}{M^{\frac{1}{m}}-1}\right),$$

(5) $$F=\frac{\rho }{\rho_{low}},\;\;M=\frac{\rho_{high}}{\rho_{low}},$$

where *ρ* is the overall resistivity of cement paste, *ρ_low_* is the resistivity of the low resistivity phase, *ρ_high_* is the resistivity of the high resistivity phase, *φ_low_* is the volume fraction of the low resistivity phase, *F* is the resistivity formation factor, *M* is the magnification coefficient between *ρ_high_* and *ρ_low_*; *φ_c_* is the percolation threshold, m is the critical exponent. This model works well to characterize various properties in the saturated cementitious system, such as the diffusion coefficient [51], water permeability [52] and resistivity [12,13,14].

**Figure 1:**
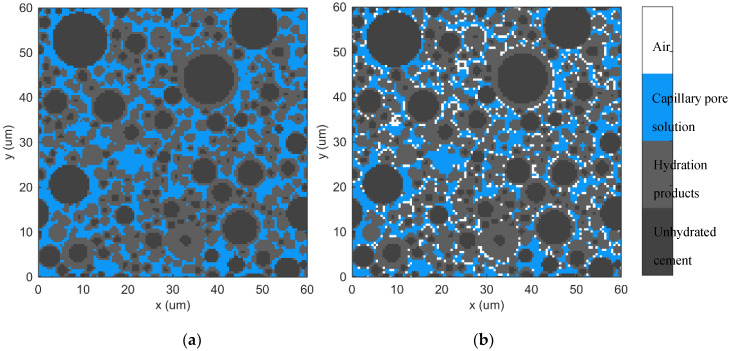
Schematic diagram of cement paste microstructure: (**a**) Saturated, (**b**) Unsaturated.

However, porosity cannot be considered as the low resistivity phase for the partially saturated system, as is shown in Figure 1. Cement paste is a complex compound consisting of liquid, gas and solid phases, including unhydrated cement particles and hydration products consisting of calcium hydroxide (CH) and C-S-H gel. Among these phases, only the capillary solution and C-S-H gel are electrically conductive but C-S-H gel has limited contribution to conductivity because its resistivity is 400 times that of pore solution [12]. It should be noted that not all pore solutions have the same contribution to conductivity. The gel pore solution, which exists in the form of small isolated capillaries, micropores and interlayer space in C-S-H gel [33,34], has limited contribution to conductivity. Because the migration of ions is blocked by the gel chain, which is the main reason why C-S-H gel has a hundred times higher resistivity than pore solution. On the contrary, the capillary pores are believed to be interconnected where they can migrate freely. Therefore, it is more reasonable to determine that the capillary solution is the main conductive phase in cementitious systems.

Another problem is how to determine the volume fraction of the capillary pore solution. In this study, the analytical cement hydration model [31] based on an NMR experiment was used. Comparing with the widely-used Power’s model [32], the model functions on the basis of continuous non-destructive monitoring of water in cement paste during hydration, and considers the density change of C-S-H. According to the analytical model, the volume fraction of gel and capillary pores (water-filled) can be calculated from Equations (6) and (7):

(6) $$\varphi_{gpor}=\{\begin{array}{ll} 0 & \;0\leq \alpha \leq \alpha_{I–II}\\ \frac{-0.799{\left(w/c\right)}^{2}+4.824(w/c)\cdot \alpha -0.793\alpha^{2}}{\left(1+3.185w/c)(0.864w/c+1.278\alpha \right)} & \;\alpha_{I–II}\leq \alpha \leq \alpha_{II–III}\\ \frac{3.185w/c-0.755\alpha }{1+3.185w/c}\geq 0 & \;\alpha_{II–III}\leq \alpha \leq 1 \end{array},$$

(7) $$\varphi_{cpor}=\frac{3.185w/c-0.755\alpha }{1+3.185w/c}-\varphi_{gpor}\geq 0,$$

where *w/c* is the water to cement ratio of cement paste, α is the hydration degree of cement paste, *φ_cpor_* is the volume fraction of capillary pore solution, *φ_gpor_* is the volume fraction of gel pore solution, $\alpha_{I–II}$ is the hydration degree at the transition from hydration regime I to II and $\alpha_{II–III}$ is the hydration degree at the transition from hydration regime II to III [31]. $\alpha_{I–II}$ and $\alpha_{II–III}$ are related with *w/c* and can be calculated from:

(8) $$\alpha_{I–II}=0.170w/c,\;\alpha_{II–III}=2.022w/c$$

From the analysis above, the capillary pore solution was regarded as the unique low-resistivity phase. According to the original definition in the general effective media equation, *φ_low_* in Equation (4) should be substituted into Equation (7) as *φ_cpor_*. Combining Equation (4), Equation (5) and Equation (7) together, the relationship between hydration degree α and resistivity *ρ* can be furtherly deduced by following equation.

(9) $$\varphi_{cpor}=\frac{3.185w/c-0.755\alpha }{1+3.185w/c}-\varphi_{gpor}=\left[\left(1-\varphi_{c}\right){\left(\frac{\rho }{\rho_{liq}}\right)}^{-\frac{1}{m}}-\varphi_{c}\right]\cdot \left(\frac{M^{\frac{1}{m}}-{\left(\frac{\rho }{\rho_{liq}}\right)}^{-\frac{1}{m}}}{M^{\frac{1}{m}}-1}\right)$$

Pore solution resistivity is constantly changing during cement hydration. The value is related to the initial alkali content, the water-cement ratio, the cement hydration age and the curing conditions [5,7,53,54]. In this study, the pore solution resistivity was calculated by an online tool developed by Bentz [55,56,57]. This model predicts the pore solution resistivity of cement paste under sealed conditions using the water–cement ratio, the alkali content (Na_2_O, K_2_O) and hydration degree. According to this model, the relationship between resistivity and hydration degree is linear and can be described by the following equations:

(10) $$\rho_{cpor}=\{\begin{array}{c} -0.0695a+0.1634\;\;\;\;w/c=0.50\\ -0.0701a+0.1480\;\;\;\;w/c=0.45\\ -0.0708a+0.1335\;\;\;\;w/c=0.40\\ -0.0717a+0.1185\;\;\;\;w/c=0.35\\ -0.0731a+0.1034\;\;\;\;w/c=0.30 \end{array}.$$

## 3. Experiment

### 3.1. Materials and Sample Preparation

ASTM Type I ordinary Portland cement with a specific gravity of 3.15 and specific surface area of 350 m^2^/kg was used in the work. The mineral composition of ordinary Portland cement tested by XRF (X-ray fluorescence spectrometer) is listed in Table 1. The ultimate hydration heat of cement is 457 kJ/kg according to the mass fraction and the hydration heat of each mineral content. Deionized water was used in the non-contact electrical resistivity measurement and isothermal calorimetry. Two kinds of cement pastes were prepared with *w/c* ratios of 0.35 and 0.45, which were denoted as P35 and P45, respectively.

### 3.2. Non-Contact Electrical Resistivity Measurement

**Figure 2:**
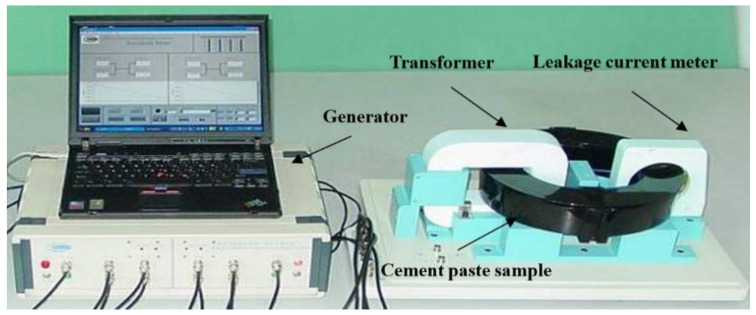
Non-contact electrical resistivity device.

The resistivity of the cement paste was obtained by a non-contact electrical resistivity measurement device. Cement and water were put into the dedicated mixer, and slowly stirred for 120 s, paused for 5 s and quickly stirred for 120 s. After mixing, 2 L of the fresh mixtures were cast in a ring-shaped mold immediately and then the mold was vibrated gently to exclude bubbles out of the paste. Afterwards, the mold was covered by a plastic lid and sealed with Vaseline to prevent evaporation of water. In this research, two temperature sensors were embedded in the specimen symmetrically to record the internal temperature of the cement paste. Moreover, another two temperature sensors were employed to monitor the ambient temperature. The data were transferred to a computer and recorded once a minute. After measurement for 72 h, the specimen was taken out of the mold. Then the height of the specimen was measured to revise the electrical resistivity measurement results. For P45, the ambient temperature was maintained at 18, 23 and 28 °C during the measurement. For P35, the resistivity was measured under a temperature of 18 °C. The non-contact electrical resistivity device can be seen in Figure 2.

### 3.3. Isothermal Calorimetry Experiment

During the isothermal calorimetry experiment, Portland cement and deionized water were put into an admix ampoule and fully mixed by a stirrer. Then the accumulated hydration heat and heat flow of cement paste were measured using a TAM Air 08 Isothermal Calorimeter, which was manufactured by TA Instruments. The isothermal calorimetry experiment was conducted for 72 h. The temperature of the cement paste sample was strictly controlled at 20 °C during the experiment process.

## 4. Experiment Results and Discussion

### 4.1. Hydration Degree

**Figure 3:**
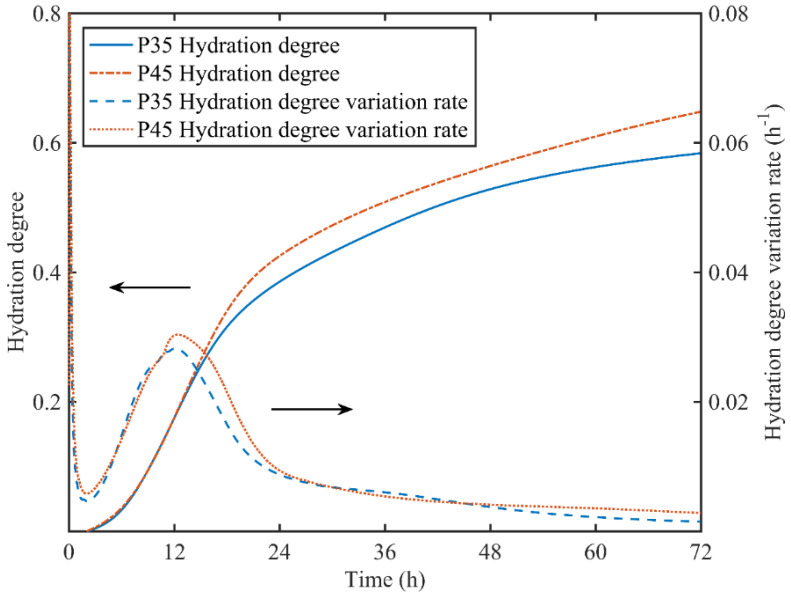
Hydration degrees and hydration degree variation rates of P35 and P45.

In this research, the hydration degree of cement paste was represented as the ratio of released hydration heat to the ultimate hydration heat calculated according to the chemical composition of cement, which is listed in Table 1. Moreover, the hydration degree variation rate was calculated from the heat flow divided by the ultimate hydration heat. The experiment results are shown in Figure 3.

In the isothermal calorimetry tests, the volume of the cement paste sample in the admix ampoule was very small, and the generated hydration heat was released to the environment rapidly. The reaction temperature of the sample maintained under the control temperature during the whole experiment process as no hydration heat was absorbed by the sample. Because the cement particles were all in sufficient contact with water at the beginning of hydration period, the hydration degree of P35 and P45 showed no obvious differences from 0 to 12 h, which can be seen in Figure 3. While afterwards, the hydration degree of P45 was higher than that of P35 due to a higher water to cement ratio. At 72 h, the hydration degrees of P45 and P35 were 0.66 and 0.58, respectively. This phenomenon is in agreement with previous research as a higher water to cement ratio means a more sufficient supply of water during the hydration process [58].

From the curve of hydration degree variation rate, the hydration process of cement pastes can be divided into three periods. In the period of 0–2 h, the heat flow is released by wetting the cement particle and the dissolution of cement particle. The decreasing hydration rate corresponds to the decrease in dissolution rate. From 2 to 24 h, the hydration rate has an evident peak corresponding to nucleation and growth reactions and phase boundary interaction reactions. Finally, the hydration process will come to an end gradually with the consumption of water. Simultaneously, the hydration rate is primarily governed by diffusion and drops close to zero from 24 to 72 h [2].

### 4.2. Electrical Resistivity

**Figure 4:**
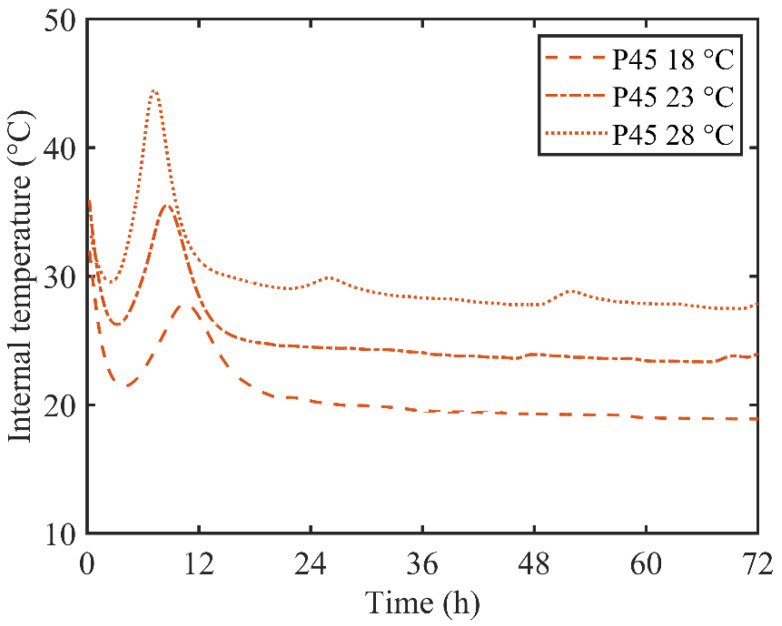
Internal temperature of P45 under different ambient temperatures.

For non-contact electrical resistivity measurement, the generated hydration heat can’t be released to the environment immediately and part of hydration heat will accumulate inside the sample. Although, the environment temperature for electrical resistivity measurement was also controlled as a constant. There was a temperature gradient from the center to the surface of the specimen. The internal temperature of P45 under different environmental conditions is shown in Figure 4.

The internal temperature actually reflects the hydration heat accumulated within the specimen during the electrical resistivity measurement. As a result, the internal temperature and the hydration degree developed similar trends. As shown in Figure 4, the internal temperature also decreased rapidly at the early age, and a temperature peak appeared in the first 24 h, followed by a gradual reduction which indicates the final dissipation of hydration heat. The internal temperature achieved its highest value as 44.6, 35.6 and 27.9 °C when the environmental temperature was 28, 23 and 18 °C, respectively. At the same time, the internal temperature reached its peak value at 7.3, 8.6 and 10.5 h for environment temperatures of 28, 23 and 18 °C, respectively. It is understandable that, as indicated in Figure 4, the high environmental temperature will promote the hydration process of cement paste, which, accordingly, will release lots of hydration heat quickly. 

**Figure 5:**
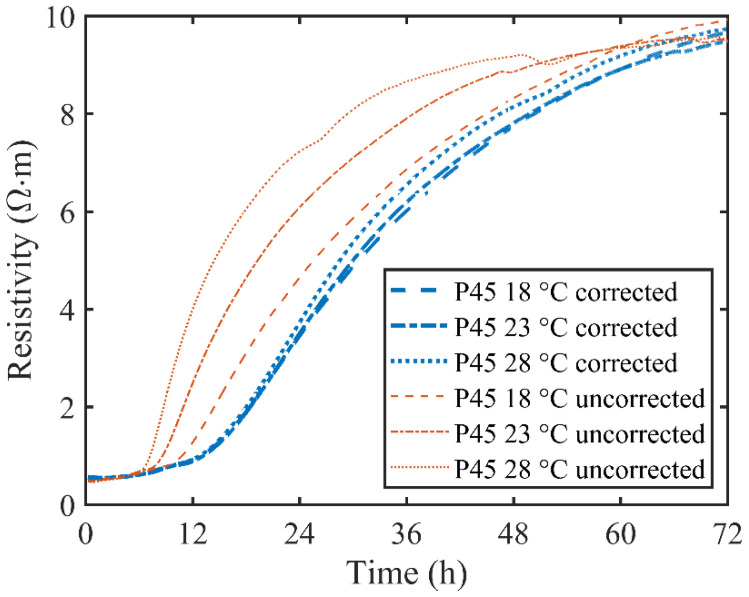
Completely and partly corrected electrical resistivity of P45 under different ambient temperatures.

**Figure 6:**
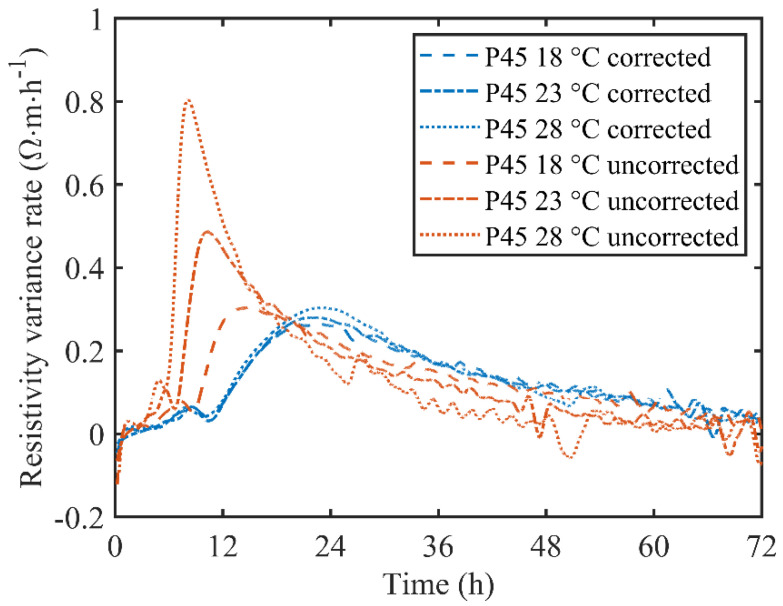
Completely and partly corrected resistivity variation rate of P45 under different ambient temperatures.

As mentioned previously, temperature can affect the resistivity of cement paste in two ways. The influence of temperature on ionic movability can be corrected utilizing a temperature correction formula. However, the influence on hydration reaction rate can’t be fully corrected from Equation (1). As a result, the resistivity and resistivity variation rate curves are still highly correlated with temperature. To essentially clarify the influence on hydration reaction rate, the completely and partly corrected electrical resistivity, as well as the resistivity variation rate, are shown in Figure 5 and Figure 6.

Although the temperature influence on ionic movability was corrected with Equation (1), the temperature had already affected the hydration process of cement paste. As shown in Figure 5, the partly corrected resistivity curves of P45 under different environmental temperature conditions are quite different from each other. A high environment temperature corresponds to a high corrected electrical resistivity, indicating a dense microstructure and a high hydration degree. This phenomenon can be furtherly explained by the development of resistivity variation rate, as shown in Figure 6. The resistivity variation rate curves show a similar trend to the internal temperature of P45. A peak can be also seen in each resistivity variation rate curve under different environmental temperatures. Moreover, a high temperature achieved a high peak value, which indicates that the electrical resistivity evolved rapidly under high temperature. Furthermore, the resistivity variation rate reached the peak quickly in the high temperature environment. The experimental results demonstrate that the influence of temperature hydration reaction rate affects the resistivity of cement paste significantly, which can’t be ignored, especially in the early stage of hydration. Therefore, this temperature influence was corrected based on equivalent age theory by substituting partly corrected electrical resistivity and internal temperature history into Equation (2).

After a complete correction, the electrical resistivity was standardized into the equivalent value in the isothermal conditions of 20 °C. Then the standardized resistivity variation rate was calculated from the derivative of the standardized resistivity with respect to time. As shown in Figure 5, the completely corrected resistivity curves of P45 at different environment temperatures were almost coincident with each other, and the resistivity at 72 h was 10.28, 9.76 and 9.60 Ω·m corresponding to 28, 23 and 18 °C, respectively. Data analysis indicated that the relative standard deviation of the resistivity at 72 h was 2.9% and the maximal relative deviation was 4%. Meanwhile, in Figure 6, the corrected resistivity variation rate curves are well coincident, and the peaks appear at 23.2, 22.9 and 22.6 h in specified values as 0.312, 0.280 and 0.259 Ω∙m∙h^−1^, accordingly. The peak value and the appearance time of all resistivity variation rate curves were both very close to each other, implying that the influence of temperature on hydration reaction rate can be corrected off by Equation (2) based on the equivalent age theory.

It can be concluded from Figure 5 and Figure 6 that a standardized treatment of measured resistivity is very necessary before further data analysis. Nevertheless, the temperature influence on hydration reaction rate is entirely neglected in some previous studies. It would cause negative consequences in two aspects: firstly, the partly corrected electrical resistivity cannot reflect the internal temperature history of samples. Strictly speaking, all the electrical resistivity results measured by non-contact resistivity devices are performed under different temperatures as different cementitious materials have unique hydration processes. Although the partly corrected resistivity characterizes the microstructure properties of cementitious materials, the essential properties of electrical resistivity are covered by internal temperature. Secondly, ignoring the temperature influence on hydration reaction rate restricts the application of a non-contact electrical resistivity measurement. It is irrational to compare the incompletely processed resistivity data with other experiment results, such as those obtained from isothermal calorimetry. Although the resistivity measurement and isothermal calorimetry test are both employed to assess the hydration process, the key time points have no similarity even if the two tests are conducted under the same environment temperature. As a result, it is the isothermal calorimetry test that is widely accepted and utilized to estimate the hydration degree of cementitious materials rather than the electrical resistivity measurement.

### 4.3. Verification of the Mathematical Model

In this study, the hydration degree was tested by isothermal calorimetry tests and then the volume fraction of capillary pore solution can be calculated by Equations (6) and (7). The electrical resistivity was measured by the non-contact electrical resistivity device and the temperature effect on resistivity was corrected carefully. The formation factor was calculated based on the corrected electrical resistivity by Equation (5). The parameters, including percolation threshold *φ_c_*, exponent parameter m, and magnification coefficient M, in the general effective media equation needed to be determined. The percolation threshold was from 0.17 to 0.20 [59,60,61,62,63] for cement paste and independent of the initial porosity or *w/c* ratio, which was selected as 0.18 in this study [64,65]. The exponent parameter m in the GEM equation was related to the geometry of the low resistivity phase with a theoretical value of 2 for cement paste [65]. Although this parameter varies from 1.4 to 2.46 for composites [49,66], it has limited effect on the calculation results [12]. In this study, the exponent parameter was limited as 1.8–2.2. The magnification coefficient was the electrical resistivity ratio of the high resistivity phase to the low resistivity phase, which varied with material and age. Garboczi and Bentz [65] indicated that the resistivity of C-S-H was about 400 times as much as that of pore solution (M = 400). While other researchers reported relatively low values of 100 [63] and 133.3 [67]. 

**Figure 7:**
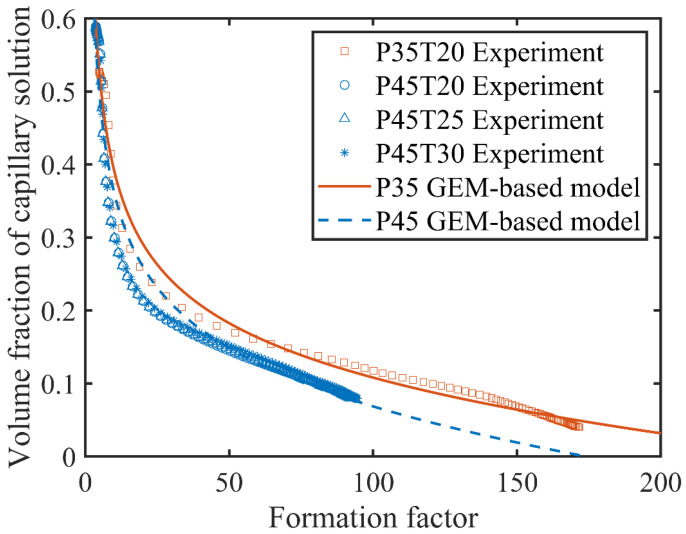
Curve fitting between volume fraction of capillary pore solution and formation factor.

In this investigation, the magnification coefficient was determined by fitting the data points of the volume fraction of capillary pore solution and the formation factor, as shown in Figure 7. Table 2 shows the results of 262.1 for P35 and 174.2 for P45, which was smaller than that of the C-S-H gel [65]. It is reasonable because the parameters were obtained based on test results during the first 72 h when C-S-H gel has a looser pore structure and higher electrical conductivity compared with the cement paste at mature age. P35 has a greater magnification coefficient than that of P45 because of a higher volume fraction of unhydrated cement particles.

**Figure 8:**
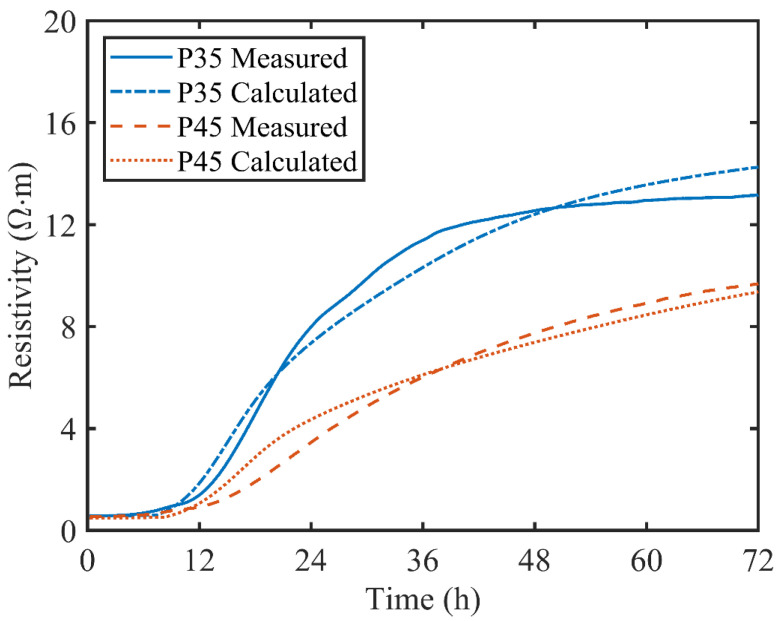
Comparison between the measured resistivity and the calculated resistivity.

Once the parameters in the general effective media equation were determined, the electrical resistivity could be predicted based on the hydration degree from isothermal calorimetry tests. In Figure 8, the predicted and measured resistivity have a good agreement overall for both P35 and P45 though there are deviations in several parts. The possible reason is that exponent parameter m and magnification coefficient M are assumed as constant in the GEM model. However, at the early hydration process, both parameters may change with evolution in the chemical composition and pore microstructure. During the dissolution stage (first 2 h) or before final setting time, the mathematical model still can provide accurate prediction results. It proves that the mathematical model can quantitatively describe the relationship between the hydration degree and electrical resistivity of cement paste, especially in the early stages of cement hydration.

## 5. Conclusions

In this work, a mathematical model was developed by considering the partially saturated state and density change of C-S-H and was well verified by the non-contact electrical resistivity test and isothermal calorimetry test. This model describes the changes in porosity and saturation state of cement compounds and the relationship between hydration degree and electrical resistivity during hydration. It has advantages in predicting the electrical resistivity of cementitious material at early age, especially for the first 48 h. The temperature correction based on the real-time temperature curve, measured by the embed temperature sensor, is recommended in resistivity measurement. This method is suitable for obtaining accurate electrical resistivity data in the early age of cement hydration.

## Figures and Tables

**Table 1:** Mineral composition and hydration heat of cement.

Mineral Composition	C_3_S	C_2_S	C_3_A	C_4_AF	Gypsum	K_2_O	Na_2_O	Total
Mass fraction (%)	65.78	7.75	6.94	8.64	6.99	0.68	0.13	97.91
Hydration heat (kJ/kg)	517	262	867	418				457

**Table 2:** The parameters of the general effective media (GEM) based model.

Mix	ϕc	M	m	R2
P35	0.18	262.1	2.184	0.9899
P45	0.18	174.2	2.003	0.9721
